# Supplementary material for: Identification of photoperiod-induced specific miRNAs in the adrenal glands of Sunite sheep (Ovis aries)
Source: Front Vet Sci. 2022 Jul 22;9:888207. doi: 10.3389/fvets.2022.888207 (PMC9354845; doi:10.3389/fvets.2022.888207)
Supplement: Supplementary file 1 [file Data_Sheet_1.ZIP › Supplementary file/miRDeep2 file.zip!/miRDeep2 file/Novel_Structure/SPLP42A2/Novel_536.pdf]

Provisional ID	:	NC_019484.2_42834
Score total	:	0.9
Score for star read(s)	:	-1.3
Score for read counts	:	0
Score for mfe	:	0.6
Score for randfold	:	1.6
Score for cons. seed	:	
Total read count	:	22
Mature read count	:	21
Loop read count	:	0
Star read count	:	1

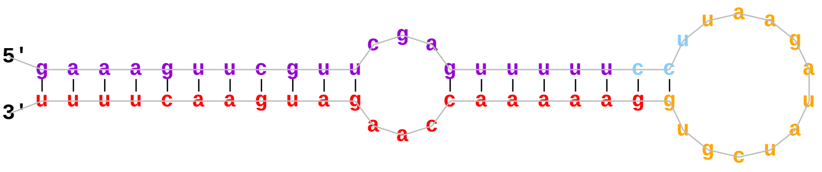

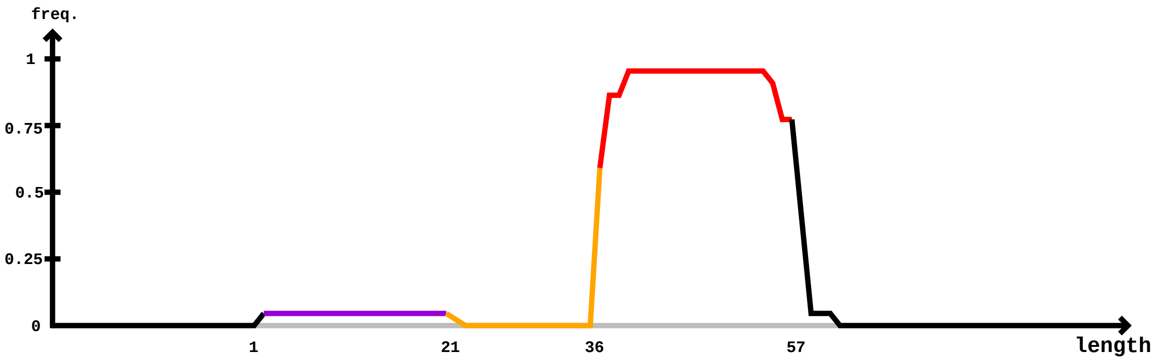

Star Mature

```
5'- uauaugaacugauuugaccagaaaguucguucgaguuuuuccuuaagauaucguggaaaaaccaagaugaacuuuuuggccaacucaauauaacugaaucacauugcugu   -3'   obs
    uauaugaacugauuugaccagaaaguucguucgaguuuuuccuuaagauaucguggaaaaaccaagaugaacuuuuuggccaacucaauauaacugaaucacauugcugu         exp
    .........(((.(((.((((((((((((((...((((((((............))))))))...)))))))))))))).))).))).......................   reads   mm   sample
    ....................gaaaguucguucgaguuuuu......................................................................   1       0    seq
    .......................................................gaaaaaccCagaugaacuu....................................   3       1    seq
    .......................................................gaaaaaccCagaugaacuuuu..................................   10      1    seq
    ........................................................aaaaaccCagaugaacu.....................................   1       1    seq
    ........................................................aaaaaccCagaugaacuuuu..................................   5       1    seq
    ..........................................................aaaccCagaugaacuuuuu.................................   1       1    seq
    ..........................................................aaaccCagaugaacuuuuuggc..............................   1       1    seq
```
